# Treatment non-adherence in pediatric long-term medical conditions: systematic review and synthesis of qualitative studies of caregivers’ views

**DOI:** 10.1186/1471-2431-14-63

**Published:** 2014-03-04

**Authors:** Miriam Santer, Nicola Ring, Lucy Yardley, Adam WA Geraghty, Sally Wyke

**Affiliations:** 1 Aldermoor Health Centre, The University of Southampton, Aldermoor Close, SO16 5ST, Southampton, UK; 2 The University of Stirling, Stirling, UK; 3 The University Of Southampton, Southampton, UK; 4 The University of Glasgow, Glasgow, UK

**Keywords:** Qualitative research, Qualitative synthesis, Child health, Medication adherence, Long-term conditions, Caregivers

## Abstract

**Background:**

Non-adherence to prescribed treatments is the primary cause of treatment failure in pediatric long-term conditions. Greater understanding of parents and caregivers’ reasons for non-adherence can help to address this problem and improve outcomes for children with long-term conditions.

**Methods:**

We carried out a systematic review and thematic synthesis of qualitative studies. Medline, Embase, Cinahl and PsycInfo were searched for relevant studies published in English and German between 1996 and 2011. Papers were included if they contained qualitative data, for example from interviews or focus groups, reporting the views of parents and caregivers of children with a range of long-term conditions on their treatment adherence. Papers were quality assessed and analysed using thematic synthesis.

**Results:**

Nineteen papers were included reporting 17 studies with caregivers from 423 households in five countries. Long-term conditions included; asthma, cystic fibrosis, HIV, diabetes and juvenile arthritis. Across all conditions caregivers were making on-going attempts to balance competing concerns about the treatment (such as perceived effectiveness or fear of side effects) with the condition itself (for instance perceived long-term threat to child). Although the barriers to implementing treatment regimens varied across the different conditions (including complexity and time-consuming nature of treatments, un-palatability and side-effects of medications), it was clear that caregivers worked hard to overcome these day-to-day challenges and to deal with child resistance to treatments. Yet, carers reported that strict treatment adherence, which is expected by health professionals, could threaten their priorities around preserving family relationships and providing a ‘normal life’ for their child and any siblings.

**Conclusions:**

Treatment adherence in long-term pediatric conditions is a complex issue which needs to be seen in the context of caregivers balancing the everyday needs of the child within everyday family life. Health professionals may be able to help caregivers respond positively to the challenge of treatment adherence for long-term conditions by simplifying treatment regimens to minimise impact on family life and being aware of difficulties around child resistance and supportive of strategies to attempt to overcome this. Caregivers would also welcome help with communicating with children about treatment goals.

## Background

Non-adherence is the primary cause of treatment failure in pediatric long-term conditions [1]. Internationally, a third to a half of all prescribed treatments are not adhered to and the rates of non-adherence amongst children and adolescents with long-term conditions is higher than amongst adults [2].

‘Adherence’ is ‘*the extent to which the patient’s behaviour matches agreed recommendations from the prescriber*’ [3]. We prefer it to the term ‘compliance’ because it includes, but does not presume, the possibility of patient involvement in the treatment decision making process. We do not use the term ‘concordance’ because it assumes a *shared* decision making process between patients and doctors [4]. Although shared decision making would ideally occur in all clinical encounters, this cannot be assumed.

Quantitative research into barriers to treatment adherence has identified a range of factors including: costs and access to treatments; complexity and demands of treatment regimen; lack of social support and depression [4]. Research into promoting treatment adherence has found that the most effective interventions are complex and include combinations of more convenient care, information, reminders, specific behavioural change techniques [3-5] and involving patients in the decision-making process [6]. Adherence interventions amongst pediatric populations also show that multicomponent interventions are most effective. [7,8] However, the effect sizes are inconsistent across studies and settings [9], effect sizes are small and more research is needed [10].

There is a recognised need for qualitative research to understand the complex behaviour of treatment adherence [3]. A systematic review and synthesis of qualitative papers on treatment adherence that focussed primarily on adults [11] found a reluctance to take medicines in general; a preference to take as little as possible; a widespread practice of personal testing of medicines, mainly for adverse effects; and patients modifying treatment regimens to make them more acceptable.

Treatment adherence in pediatric care has been less extensively studied [12], yet influences appear even more complex than in the care of adults. For example, the burden of treatment generally lies with caregivers rather than with the patients themselves. Additionally, whereas in adults the therapeutic relationship is between the medical team and the patient, in pediatric care there is a ‘therapeutic triad’ with communicative interactions between parent -professionals; child - professionals and parent - child [6].

Qualitative research is one way of better understanding the views of patients and caregivers. Whereas quantitative research and clinical trials provide strong evidence about mechanisms of adherence and effectiveness of interventions, qualitative research exploring caregivers’ experiences of treatment adherence might offer additional insights. These could inform the development of new interventions or enhance the understanding of clinicians who communicate with families regarding treatment adherence in their everyday practice. There has been no review of the qualitative literature focusing on treatment adherence in pediatrics even although there are a number of such studies which could be synthesised. We therefore conducted a systematic review and synthesis of the qualitative literature to investigate parents and caregivers’ accounts of their reasons for adherence and non-adherence to prescribed treatments in pediatric long-term medical conditions.

## Methods

The synthesis of qualitative research is an emerging field and several approaches exist [13]. We used the principles of thematic synthesis, an established approach previously used in public health [14].

### Selection criteria

Papers included in our study had to report qualitative findings, for example from interviews and focus groups, providing insight and meaning into treatment adherence or non-adherence from the perspective of parents and other caregivers (but not health professionals) of children with long-term conditions. (This topic did not need to be the primary focus of the original research studies). As our focus was on caregiver adherence, included studies had report on data from caregivers of children aged 12 or younger (studies solely reporting the views of caregivers of teenagers were excluded).

Our focus was on clinical conditions that would widely be viewed as ‘long-term illnesses’. We therefore included conditions such as asthma and diabetes but excluded behavioural, developmental and/or mental health conditions (such as autism) as well as visual and hearing impairments. Papers relating to adherence to treatments for the prevention of rejection following organ transplant were also excluded as these formed a substantial number of papers and would have led to an excessive number and heterogeneity of papers. We included studies where caregivers were given specific treatment advice and instructions but excluded studies where general advice was given. So studies reporting on parents delivering physiotherapy in juvenile chronic arthritis were included but not studies where parents were encouraging children with asthma to be more physically active. Thematic synthesis necessitates having a depth of data which can be brought together. Included papers therefore had to provide a substantial amount of data on treatment adherence or non-adherence (not just brief descriptions of these within the wider context of coping with chronic illness generally). As such, at least half the findings presented in included studies had to focus specifically on treatment adherence or non-adherence by caregivers. We excluded papers which solely reported treatment adherence in developing countries, as the barriers to treatment adherence would differ substantially in this context. To avoid misinterpreting reported findings, included papers were in English or German – languages spoken by the researchers.

### Literature search

**Figure 1:**
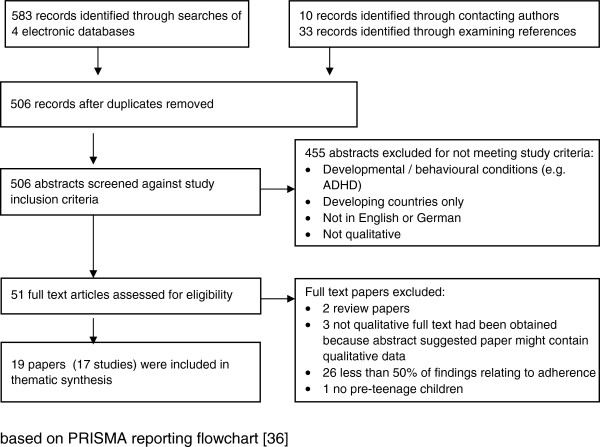
**Literature searching & screening flowchart.** Based on PRISMA reporting flowchart [16].

Four electronic databases were searched in December 2011 (Medline, Embase, Cinahl and PsycInfo) with search strategies that used both Medical Subject Headings terms and text words (see Table 1). Although there are differences in terms of meaning between adherence, concordance and compliance, all three terms were searched for to increase the sensitivity of our searches. Databases were searched from the last 15 years, as qualitative research is not well indexed prior to this date and this reflects the date of the earliest papers on this topic that we are aware of [15]. Additional papers were sought by writing to authors and examining reference lists of included papers. Titles and abstracts were initially screened and if these indicated that the paper might meet the inclusion criteria, the full text paper was retrieved and examined against our inclusion criteria. Where there was any uncertainty about inclusion, for instance if a paper provided data on treatment adherence by caregivers but of insufficient depth for synthesis, this was discussed within the research team. Further details of literature search and screening are shown in Figure 1.

**Table 1:** Sample search strategy

		**Medline (Ovid)**	**Embase (Ovid)**	**Cinahl**	**PsycInfo**
		**1996 to Dec 2011**	**1996 to Dec 2011**		
1	Patient compliance/or patient compliance.mp [mp=title, abstract, cas registry/ec number word, mesh subject heading	29572	67481	10966	687
2	Adherence.mp	48282	59237	2057	3897
3	Qualitative$.mp	80103	97242	35739	60138
4	1 or 2	70065	112359		3901
5	5 and 6	1672	2132	494	208
6	Limit 5 – all child (0 to 18 years)	331	152	86	24

Notes:

Multiple searches of the electronic databases were carried out – this is just one example. Other search strategies included using ‘concordance’ as a key word.

Searches such as the one above were then carried out in combination with common long-term conditions in children, such as asthma, diabetes, cystic fibrosis and juvenile arthritis, sickle cell anaemia, bowel disease.

### Quality of reporting

Papers meeting our inclusion criteria were quality assessed using an adapted version of the CASP quality assessment tool [17] (see Table 2). We used quality appraisal primarily to enable a critical review of each paper and to assess transparency of reporting of methods, but not as a means of excluding papers, given the debate over essential criteria for reporting qualitative studies [18]. Assessing potential papers against inclusion criteria and assessing quality was completed independently by two researchers (MS, NR, SW and AG) working independently and then collaborating to compare findings. Any differences were resolved through discussion.

**Table 2:** Quality Appraisal Criteria and Outcome of Quality Assessment of the 19 included papers

Quality assessment criteria*		**Number meeting criteria in full† (%)**	**Number meeting criteria in part (%)**	**Number not clear (%)**
1	Is this study qualitative research?	18 (95%)	1 (5%)	0
2	Are the research questions clearly stated?	16 (84%)	1 (5%)	2 (11%)
3	Is the qualitative approach clearly justified?	13 (68%)	4 (21%)	2 (11%)
4	Is the approach appropriate for the research question(s) asked?	16 (84%)	3 (16%)	0
5	Is the study context clearly described?	8 (42%)	9 (47%)	2 (11%)
6	Is the role of the researcher clearly described?	3 (16%)	10 (53%)	6 (32%)
7	Is there a clear connection to an existing body of knowledge/wider theoretical framework?	14 (74%)	3 (16%)	2 (11%)
8	Is the sampling method clearly described?	12 (63%)	5 (26%)	2 (11%)
9	Is the sampling strategy appropriate for the research question(s)?	11 (58%)	7 (37%)	1 (5%)
10	Is the method of data collection clearly described?	17 (89%)	2 (11%)	0
11	Is the data collection method appropriate to the research questions?	16 (84%)	3 (16%)	0
12	Is the method of data analysis clearly described?	14 (74%)	2 (11%)	3 (16%)
13	Is the data analysis method appropriate to the research question(s)?	6 (32%)	10 (53%)	3 (16%)
14	Are the claims made supported by sufficient evidence?	4 (21%)	13 (68%)	2 (11%)

Notes:

*Questions 1–14 incorporate the 13 criteria used by Atkins et al. [17] which is, in turn, adapted from the Critical Appraisal Skills Programme (CASP).

†Quality was assessed based on what was written in the papers. The limited word count for journal publication may mean that authors of qualitative studies omit information, particularly on methods, so what is written in a paper may not reflect the quality of the research study.

### Data extraction and analysis

Thomas and Harden [14] describe three stages in thematic synthesis: coding text; developing descriptive themes and generating analytical themes. Data were extracted from included papers in two phases. In phase 1, details about study design and participants were extracted onto a previously adapted template [19]. In phase 2, the findings and discussion from included papers relating to treatment adherence (or non-adherence) by caregivers were imported into Nvivo9 software. In order to develop descriptive themes, three reviewers independently coded this text in 10 original papers – these were chosen as they covered different conditions and provided a breadth of findings – to identify provisional themes according to meaning and content (MS, NR and SW). These three reviewers then discussed their independently derived themes and agreed a preliminary coding frame of main themes. This coding frame was then applied to data in all papers. Data were coded independently by two reviewers with any differences between coders resolved through discussion and the coding frame refined where necessary.

Once all papers were coded and data presented as descriptive themes, we needed to ‘go beyond’ the original author interpretations of their data to provide analytical themes. This was an iterative and inductive process in which we explored relationships between the different papers and long-term conditions to create new insight and meaning. This process involved extensive team discussion and reflection to refine descriptive themes and develop over-arching analytical themes derived from all included studies.

## Results

506 possible studies were assessed against our inclusion criteria with 17 studies (19 papers) finally identified as meeting our study inclusion criteria. These papers presented rich data from qualitative studies reporting caregivers views on treatment adherence or non-adherence in a range of pediatric long-term conditions (predominantly asthma, juvenile rheumatoid arthritis, cystic fibrosis, HIV, diabetes) from 423 households in five countries published over an 15 year period (1996–2011) (Table 3). Caregivers included in these studies were parents, foster parents and other relatives.

**Table 3:** Summary information on included papers

**Ref**	**Source paper**	**Condition**	**Country and setting**	**Participants**	**Data collection**
[15]	Knafl et al. 1996	Various long-term conditions	US: Recruited from 3 health centres	63 families of children age 7–14 yrs (36 diabetes, 7 renal disease, 7 asthma, 6 arthritis, 9 other)	Interviews
[20]	Bokhour et al. 2008	Asthma	US: Diverse health care settings	37 parents of 37 children age 5–12 yrs	Home interviews
[21]	Peterson-Sweeney et al. 2003	Asthma	US: Setting unclear	18 mothers of children age 2–18 yrs	Home interviews
[22]	Callery et al. 2003	Asthma	UK: Emergency room & primary care	Main caregivers of 25 young people age 9–16 yrs	Home interviews
[23]	Foster et al. 2001	Cystic fibrosis	UK: Single hospital clinic	8 mothers, 1 father of children age 10–18 yrs (8 households)	Interviews
[24]	Slatter et al. 2004	Cystic fibrosis	UK: Database of children with cystic fibrosis	17 interviews with parents of children age 3–12 years (15 households)	Home interviews
[25]	Williams et al. 2007a	Cystic fibrosis	UK: 2 hospital clinics	31 parents of 32 children age 7–17 yrs	Home interviews
[26]	Williams et al. 2007b	Cystic fibrosis	As above	As above	As above
[27]	Hammami et al. 2004	HIV	Belgium: Single hospital clinic	11 caregivers of children age 0–18 yrs	Interviews
[28]	Merzel et al. 2008	HIV	US: Treatment adherence project	14 caregivers of 15 children age 10–16 yrs	Interviews
[29]	Wrubel et al. 2005	HIV	US: Participants from research study	71 maternal caregivers (biological, foster, adoptive mothers or other female relatives) of children age 1–18 yrs	Hospital or home interview
[30]	Britton & Moore 2002	Juvenile arthritis	UK: Single hospital clinic	9 families of girls age 7–8 or 11–13 yrs	Home interviews
[31]	Sullivan-Bolyai et al. 2003a	Diabetes	US: 2 hospital clinics	28 mothers of children aged 0–4 yrs	Home interviews
[32]	Schilling et al. 2006	Diabetes	US: Participants from research studies	17 mothers and 5 fathers of 22 young people age 8–19 yrs	Home interviews
[33]	Schroder et al. 2002	Juvenile arthritis	Australia: Single hospital clinic	5 mothers of children age 3–10 yrs	Interviews
[34]	Prout et al. 1999	Asthma	UK: 2 primary care centres	9 families of children age 7–12 yrs	Varied data collection
[35]	Klok et al. 2011	Asthma	Netherlands: single hospital clinic and primary care	44 parents of children age 2–12 (34 households)	Focus groups
[36]	van Dellen et al. 2008	Asthma	Netherlands: Multicentre research	28 mothers of children age 7–17 yrs	Focus groups
[37]	Sullivan-Bolyai et al. 2003b	Diabetes	As above	As above	As above

### Findings

Overall, findings regarding caregiver treatment adherence and non-adherence were similar across conditions yet, as the impact on daily life from both the treatment and the condition varied widely, there were some notable differences between conditions. Findings that contribute to explaining treatment adherence can be summarised according to six main themes: (1) beliefs and about the condition or the treatment (2) difficulty of treatment regimen; (3) child resistance; (4) relationships within families; (5) preserving ‘normal life’; and (6) input from health professionals. Each theme is briefly described below with additional details provided in Table 4 and Table 5. We then present our analytical overview; balancing competing priorities around treatment adherence needs to be viewed in the broadest context, including preserving family relationships and promoting ‘normal life’ for the child.

**Table 4:** Example data excerpts for each theme

**Theme**	**Example data excerpts**
1a. Beliefs about the condition (assessment of symptoms, degree of long-term threat; predictability of condition and explanatory models)	Whenever he starts to come down with a cold. You know, if he has the sniffles, then I will start him. I will say okay, you should definitely be on your medication. . . . When I think that he is well enough to be taken off of the medication then I do. asthma [20]
	I do get worried about it yes. I feel very guilty, and I know, you know, we’re going to lose her, I shall lay at night thinking of all the times we didn’t do it and didn’t nag her to do it, and she’d be here now if, em, you know we had been rigid with her. cystic fibrosis [23]
1b. Beliefs about the treatment (efficacy, side effects)	I just think you hear so many things about steroids. When he was four months, he was given Prednisone, his teeth were coming out. … They got ruined . . . Some kids who get a lot of steroids, studies show that they have got hip replacements. Something that eats your bones or something. Asthma [20]
	I realize that I have the power to postpone the death of my child thanks to the medication. HIV [25]
2. Difficulty of treatment regimen	It's overwhelming. It affects everything you do even though you don't want it to. You don't want it to control your life but it does. Diabetes [15]
	As you can appreciate, if you’re putting them on at night … when she’s screaming that she can’t stand to have them on anymore too, it’s very difficult. splinting for juvenile arthritis [33]
3. Child resistance	She’s having a difficult time right now and I’m having a difficult time. She absolutely refuses to write down her blood sugars. I had taken the attitude that I wasn’t going to push and make her follow all these guidelines exactly. I don’t know if that is so good right now. It’s very difficult. Diabetes [15]
	Cause when she was small, giving her the medication didn’t have too much of a problem. She would take it. But now making sure she takes it, watching over her, standing behind, it’s really rough ‘cause she forgets. I have to be the one to remind her… sometimes she gets so careless… and I have to get rough at her, you know, about taking the medication. HIV [28]
	You end up battling with your child and getting nowhere. juvenile arthritis [30]
4. Impact on relationships within families	I felt almost cruel sometimes making her do it but I have to. juvenile arthritis [30]
	Often he says, “If you give it to me I’ll throw up.” So that night he went to bed and I didn’t give him his medication. I gave it to him the next morning and that was it. Sometimes when he’s really, really upset I don’t say anything. I just let it go. HIV [29]
	I think if you didn’t differ and you didn’t give a bit and take a bit, the children would go mentally deranged, they would, but if you were the sort of parent, and I’m sure there are, that say, right it’s 9.02 and have you had your this and have you had your that? It would crucify a child I think, I really do. cystic fibrosis [24]
5. Preserving ‘normal life’	I don’t say that much to him [about asthma]. Because I mean you have to be careful else (sic), well you can’t make them. But I try not to say much to him, you know. Because he has got to get on with his life. You know we try to let him do as much as he can and do what he can. He has got to get on with that side of his life. I mean I could make him paranoid but I think that’s why he is OK about taking his medicine. Asthma [34]
	If you’re just saying look the only thing that’s important is medication, X would say no it isn’t I want to go and have a life. cystic fibrosis [24]
	He was telling Dr A ‘I don’t like taking my medicine in school because the kids, they nosy and they bother me.’ So Dr A told me, she said well, why don’t you take your medicine at three o’clock when you come out of school, when you get home. HIV [28]
6. Input from health professionals	I didn’t actually think we were told how important the exercises were. I don’t even remember somebody saying anything. I know the importance now but if somebody had just sat down and said if only you knew how good these were, drummed it into us but they weren’t. I can remember her going to [named hospital for outpatient physiotherapy] … and she walked out of there and I thought brilliant but they never sat me down and said you’ve got to do this. juvenile arthritis [30]
	As far as this med stuff goes, having the kids making decisions, it just doesn’t work. They can’t. They’re not old enough. Their brains aren’t mature enough [laughs]. And they’re just teenagers. Teenagers can’t even make decisions about school. Easy things. My daughter and I had talked about it. And actually she doesn’t want to have to be concerned with what’s going on. That’s always been my job. And she’s not ready to have to make the decisions. She doesn’t know how to. And she’s tried to tell them that, and they’re not listening. (Adoptive mother of a 15-year-old girl) HIV [29]
	But once they talked to her and let her really know the importance of its, and that it’s for her good, she’s doing much better. HIV [28]

**Table 5:** Themes arising from included papers

**Themes**	**Asthma papers**						**Cystic fibrosis papers**				**HIV papers**			**Diabetes papers**			**Juvenile arthritis papers**		**Mixed long-term conditions**
*Reference*	*19*	*20*	*21*	*33*	*34*	*35*	*22*	*23*	*24*	*25*	*26*	*27*	*28*	*30*	*31*	*37*	*29*	*32*	*15*
Competing beliefs and concerns regarding treatment and the condition itself	✓	✓	✓	✓	✓	✓	✓	✓	✓	✓	✓	✓	✓			✓	✓	✓	✓
Difficulty, unpalatability or complexity of treatment							✓	✓	✓	✓	✓	✓	✓			✓	✓		
Child resistance		✓				✓	✓	✓		✓	✓	✓	✓		✓		✓	✓	
Preserving family relationships							✓	✓		✓	✓	✓	✓		✓		✓		✓
Preserving normality or prioritising a ‘normal life’ for the child				✓			✓		✓	✓	✓	✓		✓			✓	✓	✓
Input from health professionals		✓			✓	✓			✓	✓	✓	✓	✓		✓	✓	✓	✓	

### Caregiver beliefs about long-term conditions and treatments

This was the most commonly reported theme (noted across all 19 papers) and it had a major impact on caregiver decisions regarding treatment adherence and non-adherence. This theme consisted of two sub-themes: caregiver beliefs, concerns or fears about the condition (such as its perceived long-term threat to the child) and caregiver beliefs about the treatment (including perceived effectiveness or fear of side effects).

Thirteen papers described how caregivers attempted to weigh up beliefs about the child’s long-term condition against positive or negative beliefs about the treatment and other barriers to treatment (Table 5). For instance, in asthma, fears about potential side effects from inhaled steroids were weighed against fears of acute exacerbations [20,21], leading caregivers to carry out ‘trials’ of withholding regular medication to observe whether their child still needed them [22].

All the studies of families with children with cystic fibrosis described the tension caregivers experienced between having to overcome the many barriers to treatment adherence (especially time-consuming therapy and child resistance) against their strong belief that adherence to, at least some of, the prescribed treatments would keep their child healthy for longer [23-26]. One author described caregivers as ‘being caught between the illness, the child and the therapy’ [26]. Caregivers knew, for example, that cystic fibrosis meant their child would die prematurely but that chest physiotherapy might delay this, yet they also saw how much their child disliked and resented such treatment [26].

### Difficulty of treatment regimen

Barriers to adherence relating to specific treatment regimens was identified as a theme in approximately half of the included papers (Table 5). Caregiver reported difficulties included time-consuming or complex treatment regimens (such as chest physiotherapy for cystic fibrosis) [23-26] or unpalatable treatments or those with side effects (particularly HAART (highly active antiretroviral therapy) for HIV) [27-29] or painful treatments (physiotherapy for juvenile arthritis [30]).

Many papers reported caregivers’ descriptions of practical strategies and routines they had developed to cope with the difficulty of the treatment regimen. Some caregivers spoke positively about establishing a routine and how this could help with remembering treatments. Routines were also considered beneficial in fitting treatment regimens into family life and could help avoid child resistance developing as children came to expect their treatment as part of ‘normal’ routine.

Conversely, some caregivers perceived the rigidity of a routine as problematic, for example in children with diabetes, where caregivers described the ‘constant vigilance’ following initial diagnosis and adherence to a rigid routine gradually developed into ‘flexible adherence’ as caregivers gained confidence and were more able to adapt treatment regimens to ‘normal life’ [31]. Furthermore, some studies in cystic fibrosis [26] and HIV [28] found that caregivers believed a more flexible approach to adherence could promote the emotional well-being of the family and, therefore, the affected child.

### Child resistance

Conflicts with children over treatment adherence were widely reported by caregivers, across all conditions, as a barrier to adherence and a source of stress (Table 4 and Table 5). Some caregivers described a pattern of repetitive resistance, where the child fiercely refused most treatments leading to daily ‘battles’ and caregiver fatigue. This was particularly problematic where the treatment was aversive (unpalatable or caused adverse side-effects) or time-consuming (physiotherapy for cystic fibrosis or juvenile arthritis) as children resented the boredom of these activities.

Caregivers were not only concerned about the impact of child resistance on adherence and treatment of their condition, but they also faced dilemmas about how best to deal with it. Some caregivers were less able or willing to cope with the distress of, or dissent from, the child and these families tended to discontinue the treatment [30].

The child’s age and development influenced how caregivers viewed their responsibility for treatment adherence, their experience of child resistance and how to deal with it. A further tension was identified in diabetes and asthma, with caregivers wishing to encourage the child’s independence in managing their own care while also wishing to ensure that treatment adherence was as good as possible through parental involvement [21,32].

### Impact on relationships within families

Perceived threats and strains to family relationships was a recurring theme relating to treatment adherence particularly in papers on cystic fibrosis, HIV and juvenile arthritis (Table 5). Family relationships were considered threatened through (i) a child’s repetitive resistance to treatment leading to conflict, (ii) difficulties with handing responsibility for treatment over to older children, (iii) the child holding a different view of the treatment or condition than the parent or parents holding differing views from each other. For example, Williams et al. [26] highlighted that children may take a different view of cystic fibrosis treatment from their caregivers associating treatment with illness and infection, rather than with health and well-being [26]. Conflict between caregivers and children over treatments were sometimes directly described as resulting in non-adherence, as illustrated in Table 4 by the quote from a caregiver of a child with HIV.

### Preserving ‘normal life’

Across all the long-term conditions, authors identified how promoting ‘normal life’ for the child with a long-term condition was a parental/caregiver priority yet treatment adherence challenged this goal, particularly where treatments were time-consuming or where child resistance developed (Table 5). Time-consuming therapies, such as physiotherapy for juvenile arthritis or cystic fibrosis, presented a difficulty as this was time spent to the exclusion of other family members and other activities; some caregivers felt that the regimen had to be contained so that it did not impinge on ‘normal’ activities [26].

Highly visible therapies, such as wearing splints for juvenile arthritis [33], were also viewed as threats to ‘normal life’ by drawing attention to the child’s condition. This was particularly problematic for caregivers of children with HIV, who reported difficulties giving treatment to children in front of others who were unaware of their child’s HIV status, due to the perceived stigma of the condition [27]. Conversely, in asthma, there was evidence that inhalers were viewed as facilitating normality as they allowed children to join in activities which would otherwise have been difficult for them [34]. In diabetes, treatment was also viewed as facilitating normal life in that it kept the child well [31].

### Input from health professionals

Input from health professionals was mentioned by caregivers across all conditions as influencing their beliefs about the illness and the treatment. Health professionals were seen as a source of advice on how to overcome difficulties with the treatment regimen; or to help communicate with their child about treatment goals.

Where treatments were not observed to be immediately beneficial, a strong relationship between caregivers and health professionals appeared to have an important role in promoting treatments [25,35]. Findings from HIV studies reflected that the caregivers viewed healthcare professionals as a source of support in overcoming challenges to adherence, for instance for advice about dealing with un-palatability and gastro-intestinal side-effects [28,29]. Some caregivers felt that they were unable to get the child to fully adhere to the regimen and needed the help of health professionals, who were better able to encourage the child to implement their treatment [25,28].

Although input from health professionals was generally seen in a positive way, there were some cases where caregivers and health professionals did not share the same perspectives. For example, one study reported that caregivers felt medical staff tried to engage with their child before that child was ready to be involved in treatment decisions [29]. Also, some caregivers did not always hold the same views as their health professionals regarding a condition. For instance, caregivers did not always see asthma as long-term condition requiring constant preventative treatment or they did not perceive inhaled steroids to be a safe treatment [35,36].

### Analytical overview

Drawing together the themes from all included studies allowed us to gain an overview of the full range of factors that influence caregivers in their everyday management of treatment adherence for long term pediatric conditions. While individual authors have highlighted concerns of parents regarding balancing competing concerns about the treatment and the condition, or between child resistance and strict adherence, this overview demonstrates that this balance needs to be viewed in the widest context, including preserving family relationships and promoting ‘normal life’ for the family. Caregivers may have been implicit or explicit in their descriptions of how they attempted to reconcile their competing concerns or priorities but all experienced tensions and this complex juggling of the needs of the child and the family reflects the juggling act that caregivers carry out in everyday life.

## Discussion

This study thematically synthesised 19 papers from 17 studies in 5 countries reporting on how caregivers manage treatments in a range of long-term conditions. Our findings reveal that a wide range of factors contributed to treatment adherence (or non-adherence) in these pediatric long-term conditions. The papers we synthesised were diverse in terms of long-term conditions, types of caregivers and the age range of children cared for but one over-arching theme arising from all these studies was that caregivers sought to balance many competing concerns about the condition and its treatment in the context of everyday family life. Their ability to adhere to a treatment regimen depended on several key factors – difficulty associated with its implementation (such as treatment side-effects) and child resistance and the threat that these factors posed to family relationships and ‘normal life’ for the child and any siblings. Balancing these competing concerns was on-going for caregivers of children with long-term conditions and they worked hard to overcome challenges on a day-to-day basis. Health professionals have a key role in supporting treatment adherence in pediatric long-term conditions.

A strength of this review is that through drawing together qualitative findings on diverse pediatric long-term conditions, we were able to see patterns that may not otherwise have emerged and identify the full range of factors influencing treatment adherence and the needs of caregivers in relation to prescribed treatment regimens. Many of our findings support those of the meta-ethnography of qualitative research on treatment adherence in adults [11]. For example, both reviews found that people modify treatment regimens to make them more acceptable and to ‘fit’ with everyday life.

Our findings provide empirical support for the concept of a ‘therapeutic triad’ in pediatric adherence [6] with participants in these studies citing both their child and health professionals as important in influencing their adherence practices. Such findings can inform everyday consultations in pediatric long-term conditions. The additional complexity in the pediatric encounter of the ‘therapeutic triad’ rather than a ‘therapeutic dyad’ represents a challenge to health professionals to develop sophisticated communication strategies. For instance, health professionals may be able to assist parents and caregivers by helping the child view their treatment as enabling health and a ‘normal life’, rather than representing illness and interference [26]. Participants in these studies wished for more support from health professionals in devising simpler treatment regimens that take account of family life, seeking solutions to barriers to adherence and communicating with their child about adherence. Providing opportunities to discuss barriers to adherence before repetitive resistance develops could be a great help to caregivers.

A limitation of our review is that the participants included in these studies may not have been fully representative of less adherent families in some cases. A further limitation is that we considered only papers which included data from caregivers – we did not include papers reporting children’s views about treatment adherence. Our need to include studies with children of varying ages meant we excluded papers on other long-term conditions such as sickle cell anaemia and inflammatory bowel disease which focused on teenagers only. A synthesis of qualitative studies focusing on the views of children and young people with long-term conditions would be therefore valuable in future.

## Conclusions

In practice, treatment adherence by caregivers is the result of a complex balancing act of competing concerns including their beliefs about a condition and its treatment, managing child resistance, preserving family relationships and promoting ‘normal life’ for the family. Health professionals need to understand the complexities surrounding treatment adherence and non-adherence in order to support caregivers in developing treatment regimens that minimise impact on everyday life and family relationships. This means simplifying regimens and being prepared to discuss strategies to address or pre-empt child resistance, including communicating treatment goals to the child so far as possible.

## Competing interests

The authors declare that they have no competing interests.

## Authors’ contributions

All authors contributed to the design and analysis. MS, NR, AG and SW reviewed papers and carried out data extraction and coding. MS was responsible for drafting the paper and all authors revised the paper and approved the final version.

## Pre-publication history

The pre-publication history for this paper can be accessed here:

http://www.biomedcentral.com/1471-2431/14/63/prepub
